# Rationale and Protocol of the ETERNITY-ITA Study: Use of Etelcalcetide for Preserving Vitamin K-Dependent Protein Activity—An Italian Study

**DOI:** 10.3390/jcm13195888

**Published:** 2024-10-02

**Authors:** Maria Fusaro, Andrea Aghi, Carmela Marino, Francesca Mallamaci, Mario Plebani, Martina Zaninotto, Maria Grano, Silvia Colucci, Maurizio Gallieni, Thomas L. Nickolas, Sandro Giannini, Stefania Sella, Paolo Simioni, Alberto Bazzocchi, Giuseppe Guglielmi, Fulvia Taddei, Enrico Schileo, Maria Carmela Versace, Giovanni Tripepi

**Affiliations:** 1 National Research Council (CNR), Institute of Clinical Physiology (IFC), 56124 Pisa, Italy; 2 Department of Medicine, University of Padova, 35128 Padova, Italy; 3 Independent Researcher, 35128 Padua, Italy; andrea.aghi@gmail.com; 4 Research Unit of Clinical Epidemiology of Reggio Calabria, Institute of Clinical Physiology (IFC), National Research Council (CNR), 89124 Reggio Calabria, Italy; cmarino@ifc.cnr.it (C.M.); francesca.mallamaci@libero.it (F.M.); mcversace@ifc.cnr.it (M.C.V.);; 5 Nephrology, Dialysis and Transplantation Unit, Grande Ospedale Metropolitano, Bianchi-Melacrino-Morelli (BMM), 89124 Reggio Calabria, Italy; 6 QI.LAB.MED, Spin-Off of the University of Padova, 35011 Campodarsego, Italy; mario.plebani@unipd.it (M.P.); martina.zaninotto@aopd.veneto.it (M.Z.); 7 Department of Precision and Regenerative Medicine and Ionian Area, University of Bari, Piazza Giulio Cesare 11, 70124 Bari, Italy; maria.grano@uniba.it; 8 Department of Translational Biomedicine and Neuroscience, University of Bari, 70124 Bari, Italy; silviaconcetta.colucci@uniba.it; 9 Department of Biomedical and Clinical Sciences ‘Luigi Sacco’, Università di Milano, 20157 Milano, Italy; maurizio.gallieni@unimi.it; 10 Post-Graduate School of Specialization in Nephrology, University of Milano, 20157 Milano, Italy; 11 Division of Nephrology and Dialysis, Azienda Socio-Sanitaria Territoriale (ASST) Fatebenefratelli-Sacco, Fatebenefratelli Hospital, 20157 Milan, Italy; 12 Department of Medicine, Division of Nephrology, Columbia University, New York, NY 10032, USA; tln2001@cumc.columbia.edu; 13 Department of Medicine, Clinica Medica 1, University of Padova, 35128 Padova, Italy; sandro.giannini@unipd.it (S.G.); stefania.sella@unipd.it (S.S.); paolo.simioni@unipd.it (P.S.); 14 Diagnostic and Interventional Radiology, IRCCS Istituto Ortopedico Rizzoli, 40136 Bologna, Italy; abazzocchi@gmail.com; 15 Department of Clinical and Experimental Medicine, Foggia University School of Medicine, 71122 Foggia, Italy; giuseppe.guglielmi@unifg.it; 16 Bioengineering and Computing Laboratory, IRCCS Istituto Ortopedico Rizzoli, 40136 Bologna, Italy; fulvia.taddei@ior.it (F.T.); enrico.schileo@ior.it (E.S.)

**Keywords:** bone fractures, etecalcetide, hemodialysis, vascular calcification, vitamin K

## Abstract

**Background/Objectives:** Chronic kidney disease and mineral bone disorders (CKD-MBD) are frequently associated with an increased risk of both vascular calcifications (VCs) and bone fractures (BFs). The complex pathogenesis of VCs and BFs involves various factors such as calcium overload, phosphate imbalance, and secondary hyperparathyroidism. Key players, such as the vitamin K-dependent proteins (VKDPs) matrix Gla protein (MGP) and bone Gla protein (BGP), have pivotal roles both for VCs and BFs. The VIKI study highlighted that hemodialysis patients treated with calcimimetics had higher levels of total BGP and MGP compared to those untreated, suggesting a potential protective effect of these drugs on BFs and VCs beyond the beneficial effect of reducing PTH levels. **Methods:** ETERNITY-ITA is a multi-center, comparative effectiveness, observational, longitudinal study that will enroll 160 hemodialysis patients (80 patients treated with Etelcalcetide and 80 age- and sex-matched patients treated with calcitriol or vitamin D analogs). Nephrologists will tailor the target dose of Etelcalcetide on an individual level to achieve the KDIGO PTH target. In the Etelcalcetide-treated group, the addition of calcitriol will be allowed when required by clinical practice (for correction of hypocalcemia). **Conclusions:** This study will evaluate the real-world effect of Etelcalcetide on VKDP levels, such as BGP and MGP, at 3, 9, and 18 months from baseline. The resulting preservation of vascular and bone health will be assessed for the first time by examining aortic and iliac artery calcifications and vertebral fractures, respectively.

## 1. Introduction

Vascular calcifications (VCs) are common complication of CKD mineral and bone disorders (CKD-MBD). In CKD patients, the prevalence of aortic calcification is higher than in the general population and VCs were found to be associated with a higher prevalence of bone fractures (BFs) [1]. The pathogenesis of VC in CKD patients is multifactorial, with calcium and phosphate overload and secondary hyperparathyroidism (HPT) among the most relevant etiological factors [2]. Calcification inhibitors are also important determinants of VCs [3]. A vitamin K-dependent protein (VKDP), such as matrix Gla protein (MGP), is characterized not only by five sites of carboxylation (c-MGP) but also by the phosphorylation of three serine residues (p-c MGP, through the enzyme casein kinase) [4,5], which seem to regulate protein secretion into the extracellular environment [5]. The main function of MGP is associated with the carboxylate form, which makes it a strong inhibitor of vascular calcifications, probably due to its interaction with bone morphogenetic protein 2 (BMP-2: a powerful osteoinductive protein), which transforms vascular smooth muscle cells (VSMCs) into osteoblast-like cells [6]. c-MGP binds calcification crystals in blood vessels, forming vesicles and apoptotic bodies and preventing both calcium phosphate precipitation and the trans-differentiation of VSMCs into an osteogenic phenotype [5]. Another remarkable VKDP is bone Gla protein (BGP), which is mainly secreted by osteoblasts and in small quantities even by chondrocytes [7,8]. It has three carboxylated sites, making it active in its main bone mineralization action and allowing the interaction between the calcium-binding Gla residues and the calcium ions of hydroxyapatite, forming hydroxyapatite crystals. Indeed, BGP also acts as an inhibitor of bone mineralization, regulating mineral maturation [8].

The current paradigm for reducing the risk of vascular calcification (VC) development and progression revolves around the reduction of parathyroid hormone (PTH), calcium, and phosphorus. In particular, the recent Kidney Disease Improving Global Outcomes (KDIGO) guidelines recommend targeting PTH levels to a range between 2–9 times the upper normal limit [2]. In this scenario, Etelcalcetide emerges as a novel synthetic peptide that binds the calcium-sensing receptor-enhancing activation of the receptor itself through extracellular calcium and at the same time decreases PTH secretion in parathyroid cells [9]. Experimental studies [1] and human studies [7] have shown that oral calcimimetics use prevents vascular calcification by an MGP-mediated mechanism (increasing MGP serum levels) not seen with the use of vitamin D or its analogs.

The main aim of this study will be to assess whether Etelcalcetide, compared to vitamin D or vitamin D analog treatments, increases the levels of vitamin K-dependent proteins (VKDPs) such as bone Gla protein (BGP) and matrix Gla protein (MGP) at 3, 9, and 18 months from baseline, resulting in proper bone mineralization and inhibition of vascular calcification. The secondary aims include evaluating, at 18 months, whether treatment with Etelcalcetide decreases the progression of vascular calcification (in the aorta and iliac arteries) and vertebral fractures.

## 2. Methods

### 2.1. Study Design

**Figure 1:**
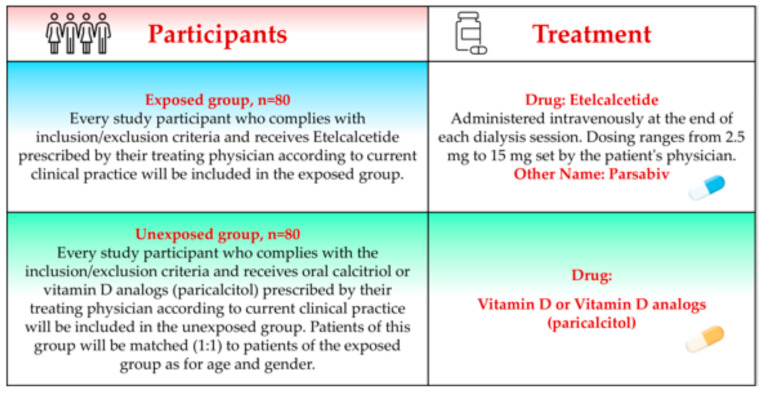
Study groups.

This is a national, multi-center, comparative effectiveness, observational, longitudinal study with no predefined interference on drug dosing by the investigators. Nineteen Italian centers will be involved. In each center, 14 patients will be screened for eligibility and about 10–12 patients are scheduled to be enrolled. This study will enroll 160 hemodialysis patients (80 patients treated with Etelcalcetide and 80 age- and sex-matched patients treated with calcitriol or a vitamin D analog, paricalcitol) (Figure 1).

**Figure 2:**
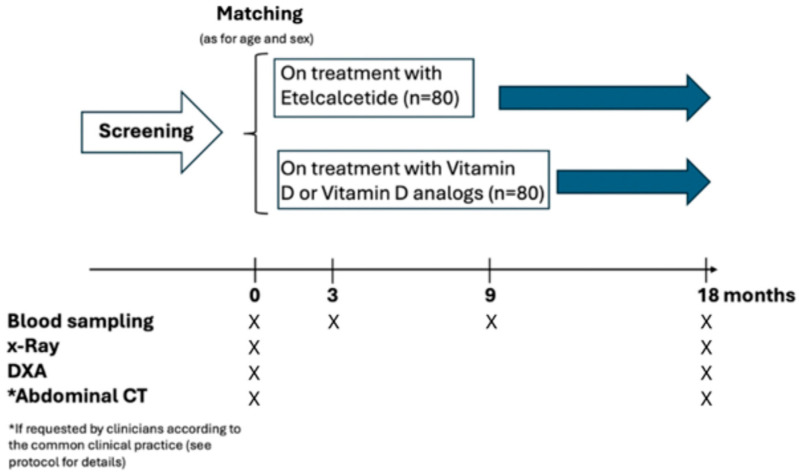
Graphical representation of the study protocol.

Given the observational nature of this study, the investigators will not recommend target goals of Etelcalcetide treatment. The treating nephrologists will tailor their target dose of Etelcalcetide on an individual-level patient basis in order to achieve the KDIGO PTH target levels. In the Etelcalcetide-treated group, the addition of calcitriol is allowed when required by current clinical practice (for correction of hypocalcemia). Blood samples for the measurement of specific biomarkers will be collected in concomitance with the routine blood sampling scheduled in each center. At baseline, all relevant demographic, clinical, and biochemical data will be collected in compliance with the privacy laws of the European Union. The study design is illustrated in Figure 2.

### 2.2. Ethical Considerations

Written informed consent will be obtained from each participant. The study is registered at clinicaltrials.gov: NCT 06352957. This study will be conducted according to the Helsinki declaration.

## 3. Study Objectives and Aims

### 3.1. Main Outcome Measures

The main outcome measure is the comparison of VKDP levels between patients treated with Etelcalcetide and those treated with vitamin D or vitamin D analogs. The longitudinal changes of the following VKDPs (measured at baseline and after 3, 9, and 18 months) will be compared between the two groups: total MGP, dephosphorylated-undercarboxylated MGP (dp-ucMGP), total BGP, and undercarboxylated BGP (ucBGP).

### 3.2. Other Outcome Measures

1. The following bone vascular marker levels will be considered: calcium, phosphate, magnesium, ALP, PTH, 25(OH)D, procollagen I intact N-terminal or P1NP, C-terminal telopeptide or CTX, tartrate-resistant acid phosphatase 5b or TRAP 5bC-Terminal to Intact, bone-specific alkaline phosphatase (BSAP), fibroblast growth factor 23 or cFGF23 and iFGF23, klotho and soluble α-klotho, sclerostin and bioactive sclerostin, DKK1, fetuin-A, zinc, and irisin.

The analysis will assess whether Etelcalcetide will affect the levels of the above-mentioned biomarkers over time.

2. Evaluation of serum calcification propensity using the T50 test [10,11].
3. Changes from baseline of the following anemia marker levels are considered: hemoglobin (Hb), hematocrit (Ht), plates (PLTS), reticulocytes, iron, ferritin, transferrin, and transferrin saturation. This is to test whether 3, 9, and 18 months of treatment with Etelcalcetide improves anemia status (e.g., reduced EPO and/or iron doses).
4. Changes from baseline of the following dialysis routine biomarkers: albumin, KT/V, aluminum, C-reactive protein (CRP), cholesterol, triglycerides, cholesterol HDL, and cholesterol LDL.

The analysis will assess whether Etelcalcetide will affect the levels of the above-mentioned biomarkers over time.

5. Changes from baseline prevalence VCs (aorta and iliac arteries) measured using a lateral dorsal lumbar spine X-ray [time frame: 18 months].

To test whether 18 months of treatment with Etelcalcetide reduces VC progression.

6. Changes from the baseline prevalence of vertebral fractures (VFs, quantitative vertebral morphometry using dedicated software, Optasia Medical SpineAnalyzer 4.0, version 4.0.2.19) measured using a lateral dorsal lumbar spine X-ray [time frame: 18 months].

To test whether 18 months of treatment with Etelcalcetide reduces VF progression.

7. Changes from baseline total hip, femoral, and neck bone mass density (BMD), measured by dual-energy X-ray absorptiometry (DEXA), including a trabecular bone score (TBS) when available. To test if 18 months of treatment with Etelcalcetide improves BMD and TBS.
8. To assess the relationship of bone vascular biomarkers and clinical outcomes: VFs and VCs.
9. To compare a novel quantitative computer-assisted scoring method for vascular calcifications with a three-dimensional assessment from CT data [12,13].
10. To evaluate the effect of Etelcalcetide on cardiovascular events and all-cause mortality.
11. To evaluate the safety of Etelcalcetide and drug interactions.

### 3.3. Inclusion Criteria

1. Patient has provided informed consent.
2. Patient is 18 years of age or older of both sexes.
3. Patients receiving maintenance HD three times per week (Kt/V >1.2).
4. Parathyroid hormone concentrations >500 ng/l at screening, or if parathyroidectomy is planned or expected, Ca >8.3 mg/dL.
5. Will be considered in the exposed group:
  - patients who have started Etelcalcetide within 1 month of study enrolment;
  - patients naïve to intravenous calcimimetics use;
  - patients who have suspended oral calcimimetics for at least 1 month;
  - patients who are not responding to or non-compliant with treatment with calcitriol.
6. In the unexposed group, patients being treated with calcitriol or vitamin D analogs and who are age- (±2 years) and sex-comparable (matching) to those in the exposed group will be considered.
7. Native vitamin D can be used in both groups and should be administered to target a 25(OH)D level >30 ng/mL.
8. Dialysate calcium concentration must be stable for at least 4 weeks prior to screening laboratory assessments.
9. Patient must have severe HPT as defined by two laboratory screening pre-dialysis serum PTH values >500 pg/mL, measured on two consecutive lab checks prior to entering the study. PTH levels should be standardized as reported elsewhere [14].
10. Total alkaline phosphatase greater than the normal range, or even within the normal range but if greater than the tertile of the reference range for the assay.
11. Patients will be eligible only if they will show at least moderate aorta VCs [15] and/or iliac artery VCs and at least a mild VF [16,17].

### 3.4. Exclusion Criteria

1. Previous treatment with oral calcimimetics (cinacalcet) must have been suspended for at least 30 days. Recent start of calcimimetics (Etelcalcetide) is acceptable, but patients are excluded if the treatment lasts for more than 1 month.
2. Patients who received a bisphosphonate, denosumab, or teriparatide during the 12 months prior to screening.
3. Patients who underwent parathyroidectomy in the 6 months before the start of the study or if the procedure is scheduled soon.
4. Scheduled kidney transplant during the study period or anticipated living donor evaluation within three months of recruitment.
5. Patients with unstable medical conditions based on medical history, physical examinations, and routine laboratory tests, or otherwise deemed unstable in the judgment of the Investigator.
6. Having metabolic bone diseases not related to the kidney (i.e., Pagets, Osteogenesis Imperfecta).
7. With severe untreated hyperthyroidism.
8. With malignancy within the last 3 years (except non-melanoma skin cancers or cervical carcinoma in situ).
9. Patients pregnant or nursing.
10. Patients with Long QT Syndrome.
11. Patients who are unlikely to be available to complete all protocol-required study visits or procedures, and/or to comply with all required study procedures to the best of the patient’s and Investigator’s knowledge.

## 4. Statistical Considerations

### 4.1. Sample Size

One hundred and sixty patients on hemodialysis are needed to address the study hypothesis. The sample size was established on pragmatic grounds by considering the statistical models that will be applied to address the study hypothesis [i.e., the linear mixed models (LMM) analysis or generalized estimating equations (GEE)]. In this case, a total of 640 observations over the time period will be collected in 160 patients, thus ensuring adequate power to adjust for major potential confounders.

### 4.2. Data Analysis

Normally distributed variables will be summarized as means and standard deviations, non-normally distributed variables as medians and interquartile ranges, and categorical data as absolute numbers and percentages. At baseline, the between-groups comparisons will be performed using the independent *t*-Test, the Mann–Whitney U Test, or the Chi-squared test, as appropriate. The between-groups comparisons of repeated measurements of biomarkers (as continuous variables) over time will be performed by linear mixed models (LMM) or generalized estimating equations (GEE). No imputation method of missing values will be applied. In these analyses, the effect of the exposure to Etelcalcetide in treated patients versus those untreated will be preliminarily investigated by including the group variable, the time, and the interaction term between the group variable and time. The effect of potential confounders on the effectiveness of Etelcalcetide on the study outcomes will be further investigated by adjusting for all potential confounders, including medications, by multiple regression models. Sensitivity analysis by applying the propensity score approach will be also performed. The primary analysis will be conducted considering the patients as belonging to the treatment arm they had at the beginning of observation. A secondary analysis will also be performed taking into account any cross-over. Survival analyses will be performed using the Kaplan–Meier and Cox regression methods. All statistical analyses will be performed using a standard statistical package (STATA 16, StataCorp. Stata Statistical Software. College Station, TX, USA).

## 5. Laboratories and Instrumental Data

The following laboratory testing will be performed:

- As part of current clinical practice, the following routine biomarkers, such as calcium, phosphate, magnesium, ALP, PTH, 25(OH)D, Hb, Ht, PLTS, reticulocytes, Fe, ferritin, transferrin, and transferrin saturation, will be measured in each participating center.
- Specific bone vascular markers (which will be measured in a centralized laboratory) considered are total MGP and BGP, dp-ucMGP, ucBGP, procollagen I intact N-terminal or P1NP, C-terminal telopeptide or CTX, tartrate-resistant acid phosphatase 5b or TRAP 5bC-Terminal to Intact, bone-specific alkaline phosphatase (BSAP), fibroblast growth factor 23 or cFGF23 and iFGF23, klotho and soluble α-klotho, sclerostin and bioactive sclerostin, DKK1, fetuin-A, and Zinc.

### 5.1. Instrumental Data

#### 5.1.1. X-ray

According to the current indications of good clinical practice and guidelines, at baseline, the patients will undergo a lateral dorsal lumbar spine X-ray using dedicated software (Calcify 2D [13]) to evaluate vascular calcifications (VCs: aorta and iliac arteries) [12,13,14,15] and VFs, where vertebral fractures will be identified if the height of the vertebral body is reduced by at least 20% or 4 mm, according to Genant et al. [16]. Furthermore, a lateral dorsal lumbar spine X-ray will be performed at the end of follow-up at 18 months. Both VCs and VFs will be assessed independently by two physicians who are blinded to the patient’s clinical characteristics.

#### 5.1.2. DXA

According to the current indications of good clinical practice and guidelines [2], at baseline, measurements of total hip, femoral, and neck bone mineral density (BMD) will be assessed by dual-energy X-ray absorptiometry (DXA), including a trabecular bone score (TBS) when available. Furthermore, DXA and TBS will be performed at the end of follow-up at 18 months.

#### 5.1.3. Abdominal CT

If the physician, in the case of the presence of moderate/severe aorta VCs associated with co-morbidity with a resulting higher risk of cardiovascular and mortality events, prescribes an abdominal CT, these findings will be included in the study as data reflecting common clinical practice. In addition, a subset of patients with aorta calcifications confirmed by X-rays will undergo CT scans of the lumbar spine. According to [12], calcification will be considered to be present if an area of ≥1 mm^2^ displays a density of ≥130 Hounsfield units. The AAC score will be calculated from the takeoff of the renal artery to the bifurcation of the aorta into the common iliac arteries. The cross-section of the abdominal aorta on each slice will be divided radially into 12 segments. The abdominal aortic calcification index (ACI) will be calculated as follows: ACI = (total score for calcification in all slices)/12 × 1/(number of slices) × 100 (%). Semi-quantitative measurement of AAC on lateral X-rays will be conducted independently by two physicians who are blinded to the patient’s clinical characteristics. The general description of this study (including the types of clinical examinations and blood sampling over time) is given in Figure 2.

## 6. Preliminary Results

In a previous paper by us [7], we measured BGP and MGP levels and checked for the presence of vertebral fractures and vascular calcifications using a lateral dorsal lumbar spine X-ray through dedicated software (Optasia Medical SpineAnalyzer 4.0, version 4.0.2.19) to evaluate VFs, which were identified by a height vertebral reduction of at least 20% or 4 mm, according to Genant et al. [16]. In the same X-ray, we evaluated vascular calcifications (VCs: aorta and iliac arteries) [7] in 387 hemodialysis patients enrolled in the VIKI study. Total BGP levels were found to be twice as high in patients on calcimimetics compared to those untreated with this drug class (290 vs. 158.5 mcg/L, *p* < 0.0001), whereas total MGP levels were 19% higher in patients on calcimimetics (21.5 vs. 18.1 mcg/L, *p* = 0.04) than in the untreated group. Median total BGP levels were 29% lower in patients with one or more vertebral fractures (151 vs. 213 mcg/L, *p* = 0.0091) and 36% lower in patients with vascular calcifications (164 vs. 262.1 mcg/L, *p* = 0.0003). In non-survivors, median BGP and MGP levels were lower, but only the difference in MGP levels reached statistical significance (152 vs. 191 mcg/L, *p* = 0.20 and 15.0 vs. 19.7 mcg/L, *p* = 0.02, respectively). In another paper by our group [18], we assessed the prevalence of vitamin K deficiency and the relationship between vitamin K status, vertebral fractures, and vascular calcification in the same patient cohort. Overall, we found a relatively high proportion of patients with deficiencies in MK7 (35.4%), vitamin K1 (23.5%), and MK4 (14.5%). Of note, vitamin K1 deficiency was the strongest predictor of vertebral fractures (odds ratio [OR], 2.94; 95% confidence interval [CI], 1.38–6.26). MK4 deficiency was a predictor of aortic calcification (OR, 2.82; 95% CI, 1.14–7.01), whereas MK5 deficiency appeared to protect against it (OR, 0.38; 95% CI, 0.15–0.95). MK7 deficiency was a predictor of iliac calcification (OR, 1.64; 95% CI, 1.03–2.60). The presence of vertebral fractures was also a predictor of vascular calcifications (OR, 1.76; 95% CI, 1.00–3.08).

## 7. Discussion

In addition to its known suppressive effect on PTH, Etelcalcetide has demonstrated additional benefits in recent studies, both experimental and human. In rats, it has been shown to preserve cortical bone structure and bone strength by lowering PTH in subtotal Nx rats with established SHPT [19,20]. In humans, it has been found to improve areal bone mineral density and trabecular quality in the central skeleton, always by lowering bone turnover without affecting bone material properties, thereby reducing the risk of fractures [21,22]. A recent meta-analysis comparing various calcimimetics has shown that Etelcalcetide is the most efficacious calcimimetic agent for lowering serum PTH levels but highlighted side effects such as hypocalcemia, nausea, and vomiting. Cinacalcet ranked worst for nausea and had somewhat lower effectiveness, while Evocalcet (in use in Asian populations) had lower effectiveness for decreasing PTH levels but was associated with fewer adverse effects [23].

Ca^2+^-sensing receptors (CaSRs) are broadly expressed in the vascular system, including perivascular neurons, vascular endothelial cells (VECs), and vascular smooth muscle cells (VSMCs). Prolonged serum calcium is thought to reduce the sensitivity and expression of CaSR in arteries, thereby increasing intra-vascular Ca^2+^ concentrations through a negative feedback loop. Excess intravascular calcium gradually deposits in VSMCs and may in part lead to the transformation of VSMCs into osteoblast-like cells, leading to the formation of vascular calcification [24]. In experimental studies, overexpression of the CaSR reduced the deposition of calcium in human aortic smooth muscle cells (HASMCs) [24]. In uremic rats with secondary HPT treated with calcimimetics (R-568 or AMG 641), compared to the untreated mice, Rodriguez et al. reported that VCs were prevented and mortality was reduced in excess of what was expected from a reduction in calcium and phosphorus alone [25]. They have also ascribed a protective role to calcimimetics as they play a role in the expression of proteins that prevent the development of VCs, in particular highlighting an increase in MGP and a resulting reduction in osteoblastic gene expression markers such as BMP2 [7,25,26]. To confirm these findings, our group conducted a secondary analysis of the Vitamin K Italian (VIKI) study to assess the prevalence of vitamin K deficiency in hemodialysis patients [18] and to evaluate associations between drug consumption and VKDP levels in 387 hemodialyzed patients [7]. A previous study by us reported, in patients managed with calcimimetics compared to without calcimimetics, that total BGP levels were twice as high (290 vs. 158.5 mcg/L, *p* < 0.0001) and that total MGP levels were 19% higher (21.5 vs. 18.1 mcg/L, *p* = 0.04) [7,18]. Furthermore, Yu et al. showed lower aorta calcium content in uremic rats treated with Etelcalcetide than in paricalcitol-treated rats [27]. The precise mechanism by which calcimimetics enhance VKDP activity remains unclear.

Furthermore, recent studies have also shown that irisin, the hormone-like myokine crucial for muscle-bone communication [28], was significantly lower in hemodialysis patients compared with control subjects. Most importantly, within the group of hemodialysis patients, irisin was significantly lower in those with VCs compared to patients with no calcification [29].

### 7.1. Statistical and Methodological Issues

The study protocol rests on a solid longitudinal design, contemplating repeated measurements of specific biomarkers over time. When dealing with an observational study of effectiveness and with LMM/GEE (see Statistical Analysis) having repeated measurements over time as dependent variables, the estimation of the sample size mainly depends on the number of potential confounders to be included in the models rather than on a predefined effect of the drug. In this case, a total of 640 observations over the time period are expected to be collected [i.e., 80 patients x four longitudinal visits including baseline (n = 320 observations) in the exposed group and 80 patients x four longitudinal visits including baseline (n = 320 observations) in the unexposed group]. This high number of observations will allow us to adjust the effect of Etelcalcetide versus calcitriol or vitamin D analogs on study endpoints for both baseline and longitudinal potential confounders, thus obtaining robust and precise effect estimates. Furthermore, linear mixed models and generalized estimating equations analyses are robust methods for dealing with various patterns of missingness, an issue of particular relevance in studies of comparative effectiveness. Furthermore, since this is not an interventional study, the observational nature of the protocol will be guaranteed by inviting treating nephrologists to tailor the appropriate dosage for each patient individually, aiming to reach the KDIGO PTH target level as in everyday clinical practice. In the group receiving Etelcalcetide, calcitriol may also be added as needed according to the standard clinical approach to address hypocalcemia.

### 7.2. Dissemination Plan

The target audience to disseminate the results of the ETERNITY-ITA study includes healthcare professionals (nephrologists, endocrinologists, and general practitioners), patients suffering from chronic kidney disease and related conditions, pharmaceutical companies involved in the production of Etelcalcetide and calcitriol, and policy-makers and regulatory bodies involved in healthcare decision-making. The dissemination channels include scientific journals, national and international meetings, and online platforms such as ResearchGate, LinkedIn, and professional forums.

### 7.3. Limitations

As an observational study of comparative effectiveness, ETERNITY-ITA can provide valuable insights into how treatments perform in real-world settings. However, the observational design comes with limitations that can affect the reliability and validity of the findings, such as those due to confounding. However, such problems will be minimized by collecting data and adjusting for major potential confounders that may interfere with the pathway between treatments and outcome variables. Notwithstanding such an approach aimed at controlling for confounding variables, it is not possible to completely exclude that there may be unmeasured confounders that remain unaccounted for, leading to residual confounding and potentially biased estimates of treatment effects.

## 8. Conclusions

The clinical remarkable significance of this study is to provide first-time data on the ability of Etelcalcetide in real life to prevent increased VKDP levels, alone or in association with other vascular bone markers, such as skeletal fragility and vascular calcifications, in hemodialysis patients, with a resulting decrease in morbidity and mortality.

## Data Availability

Data are contained within the article.
